# Complementary Role of the Computed Biomodelling through Finite Element Analysis and Computed Tomography for Diagnosis of Transcatheter Heart Valve Thrombosis

**DOI:** 10.1155/2018/1346308

**Published:** 2018-10-22

**Authors:** Francesco Nappi, Laura Mazzocchi, Sanjeet Singh Avtaar Singh, Simone Morganti, Jean-Louis Sablayrolles, Christophe Acar, Ferdinando Auricchio

**Affiliations:** ^1^Department of Cardiac Surgery, Centre Cardiologique du Nord de Saint-Denis, Paris, France; ^2^Department of Civil Engineering and Architecture, University of Pavia, Pavia, Italy; ^3^Department of Cardiothoracic Surgery, Golden Jubilee National Hospital, Glasgow, UK; ^4^Department of Electrical, Computer, and Biomedical Engineering, University of Pavia, Pavia, Italy; ^5^Department of Imaging, Centre Cardiologique du Nord de Saint-Denis, Paris, France; ^6^Department of Cardiac Surgery, Hopital La Pitie Salpetriere, Paris, France

## Abstract

**Introduction:**

The TAVR procedure is associated with a substantial risk of thrombosis. Current guidelines recommend catheter-based aortic valve implantation for prohibitive-high-risk patients with severe aortic valve stenosis but acknowledge that the aetiology and mechanism of thrombosis are unclear.

**Methods:**

From 2015 to 2018, 607 patients with severe aortic valve stenosis underwent either self-expandable or balloon-expandable catheter-based aortic valve implantation at our institute. A complementary study was designed to support computed tomography as a predictor of complications using an advanced biomodelling process through finite element analysis (FEA). The primary evaluation of study was the thrombosis of the valve at 12 months.

**Results:**

At 12 months, 546 patients had normal valvular function. 61 patients had THVT while 6 showed thrombosis and dislodgement with deterioration to NYHA Class IV requiring rehospitalization. The FEA biomodelling revealed a strong link between solid uncrushed calcifications, delayed dislodgement of TAVR and late thrombosis. We observed an interesting phenomenon of fibrosis/calcification originating at the level of the misplaced valve, which was the primary cause of coronary obstruction.

**Conclusion:**

The use of cardiac CT and predictive biomodelling should be integrated into routine practice for the selection of TAVR candidates and as a predictor of negative outcomes given the lack of accurate investigations available. This would assist in effective decision-making and diagnosis especially in a high-risk cohort of patients.

## 1. Introduction

Previous generations of catheter-based aortic valve implantation consisted of conventional stented xenograft prostheses with a stent characterized by a specific material. Transcatheter Aortic Valve Replacement (TAVR) is a popular option for prohibitive-high-risk patients with symptomatic aortic valve stenosis. Newer generations of biological valves are preferred because they provide enhanced hemodynamic performance and potentially greater durability [1–9]. These valves have an increased longevity due to the lower mechanical stress imposed on the leaflets [9–17], without requiring long-term anticoagulation; however, structural valve degeneration (SVD) remains a concern, affecting the longevity of the stented xenograft prostheses [18–23]. Nevertheless, freedom from oral anticoagulation related complications and excellent quality of life support the use of biological materials [24–29].

These benefits are less demonstrable when the most articulated armamentarium of TAVR is implanted in the aortic root. As a matter of fact, two fundamental obstacles could obscure the debate on the extensive indication of TAVR proposed in the guidelines: thrombosis of catheter-based aortic valve and early structural valve degeneration SVD [30–38], two extremes of aortic surgical bioprosthesis dysfunction [39, 40].

Currently, successful planning of the catheter-based aortic valve implantation has been supported by the use of computerized tomographic images that provide crucial information concerning the aortic root anatomy as well as the peripheral vessel access. However, CT scan images are unable to predict complications after TAVR procedure based on calcium index scores. There is clinical equipoise between TAVR and conventional surgery in intermediate-risk patients [30] with a possibility of extension to low-risk patients scheduled for surgery once concerns regarding long-term durability of TAV are resolved. Presently, the mechanisms for progression to thrombosis are not well understood and cannot be directly measured using CT imaging. The pathophysiology of bioprosthetic thrombosis involves the leaflet cusps and regions of increased calcium deposition that are persistent after TAVR deployment [41, 42]. Finite element analyses (FEAs) however may play a role in determining outcomes. We applied the FEA to catheter-based aortic valve procedure and performed predictive biomodelling to investigate potential mechanisms of failure and thrombosis formation. Finite element (FE) models require accurate 3-dimensional (3D) geometry reconstruction derived from CT scans in zero-stress state, material properties, and physiologic loading conditions.

Our goal was to study the mechanism of thrombus formation and device dislodgement in the presence of persistent calcific blocks with varying calcium score indices in our series of prohibitive-high-risk patients who underwent catheter-based aortic valves intervention.

## 2. Methods

### 2.1. Study Design

We studied patients with severe aortic valve stenosis who underwent either self-expandable or balloon-expandable catheter-based valve replacement. The study was stratified to ensure ongoing equivalence between evaluation of CT scan and biomodelling through finite element analysis (FEA) aiming to develop a biomechanics model to prevent thrombosis of TAVR and dislodgment of the device. A cohort of 607 catheter-based aortic valve recipients were used to perform three types of biomechanical models. The endpoint was evidence of transcatheter heart valve (THV) thrombosis (THVT) and delayed TAVR malposition at 12 months.

### 2.2. Patients

**Figure 1:**
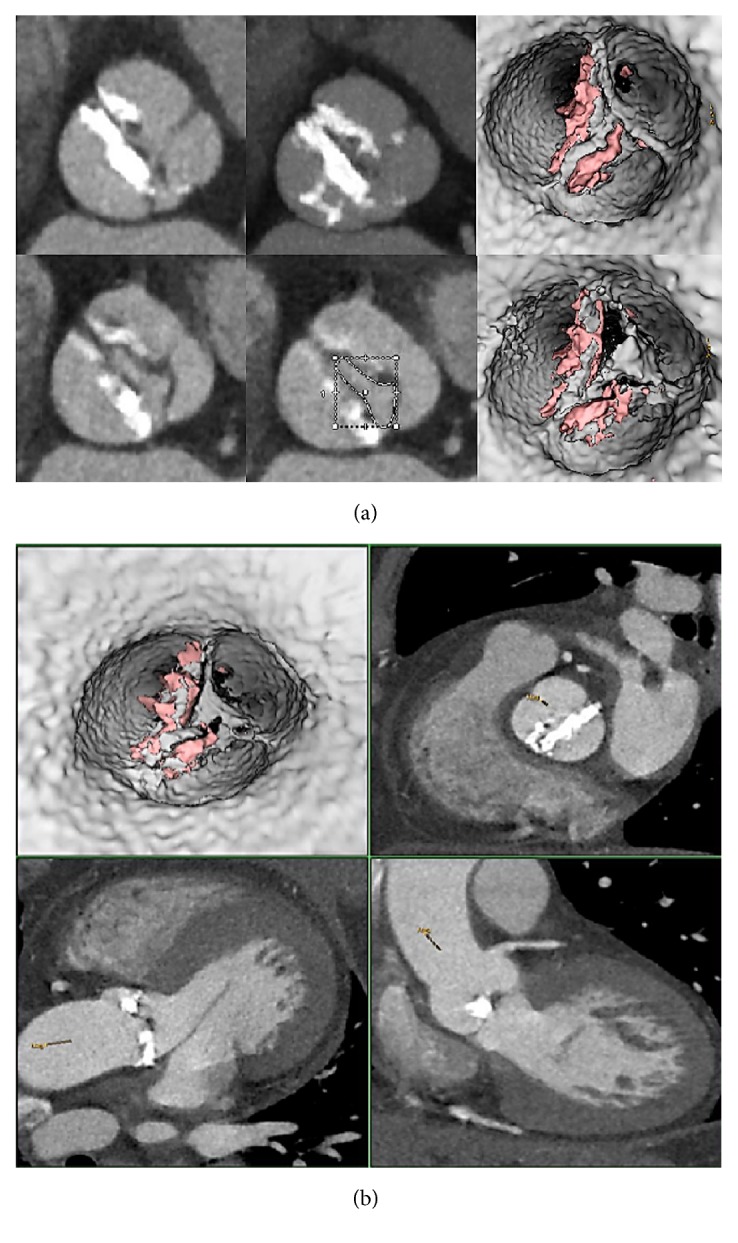
320 CT scan shows refractory calcifications on aortic leaflets of a prohibitive high-risk patient who undergo TAVR. The procedure performed was as follows: one beat full cardiac cycle (0-100%) acquisition DLP = 459 mGy/cm for functional aortic valve assessment, morphological aortic valve study, and anatomical AVA determination.

**Figure 2:**
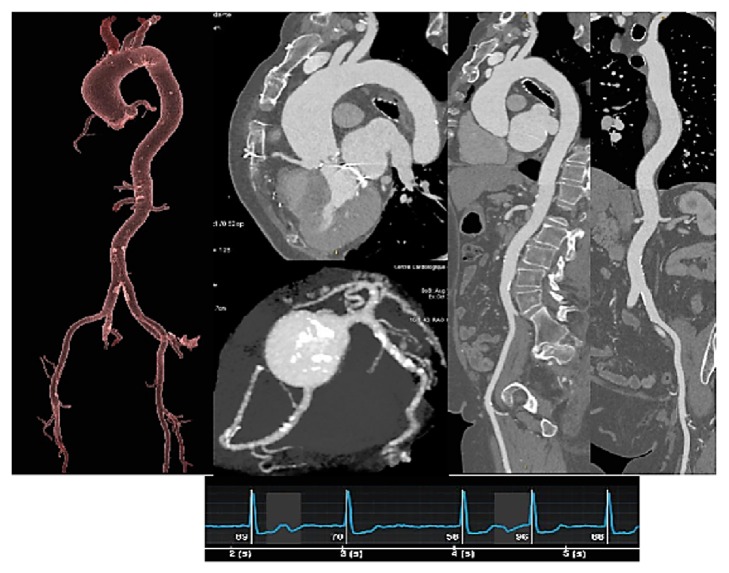
TAVR planning.

The target population was adults with severe symptomatic aortic valve stenosis eligible for TAVR procedure who were deemed prohibitive/high risk with a high comorbidity score (Videos 1 and 2). Severe aortic valve stenosis was assessed using resting transthoracic echocardiography on the basis of integrative criteria that was verified by an independent core laboratory. Severe aortic valve stenosis was defined as Vmax >/=4 m/sec or mean ΔP >/=40 mm Hg with AVA ranging between 0.6 and 1.0 cm2. All patients had ejection fraction <50% and were studied at Centre Cardiologique du Nord using transthoracic and transesophageal echocardiography (TTE and TEE) alongside computed tomography scan (CT scan). Quality of 2D ultrasound technique was ensured by Philips IE33-X6 Matrix with high resolution for specific measurements of aortic valve leaflets and especially with regard to the free margin length of leaflets. CT scans were performed using GE 230 multislice. The characteristics of the CT imaging procedure are described in Table 1; Figures 1 and 2. From 2015 to 2018, we screened a population of 607 patients eligible for this study. 546 patients had no evidence of THVT while 61 patients had thrombus formation of their catheter-based aortic valve. In six patients with THVT and dislodgment of device, the calcium score index was lower than 2359 AU; with one patient who presented with thrombosis of the right and left coronary ostia having a very low calcium score index (820 AU). The echocardiography core laboratory and CT scan confirmed the diagnosis of THVT and delayed device malposition.

### 2.3. Patients Group and Description of Biomodelling

**Figure 3:**
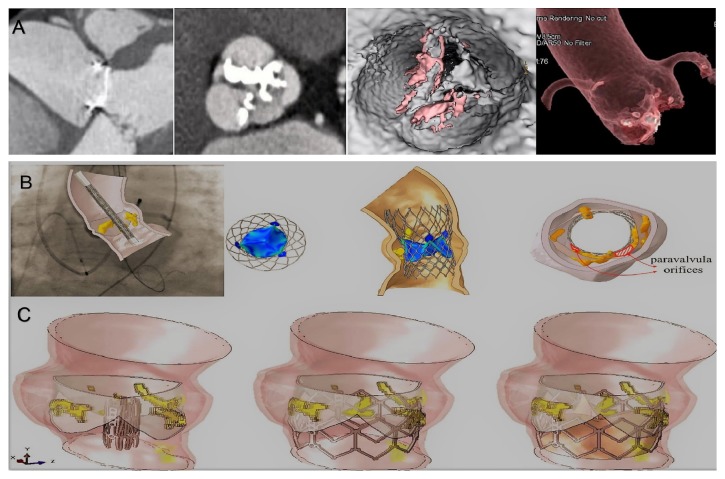
Biomodelling of the TAVR without THVT and dislodgment. Panel A: preoperative Ct scan. Panel B: biomodelling of CoreValve 26 mm. Panel C: biomodelling of Sapien XT.

**Figure 4 fig4:**
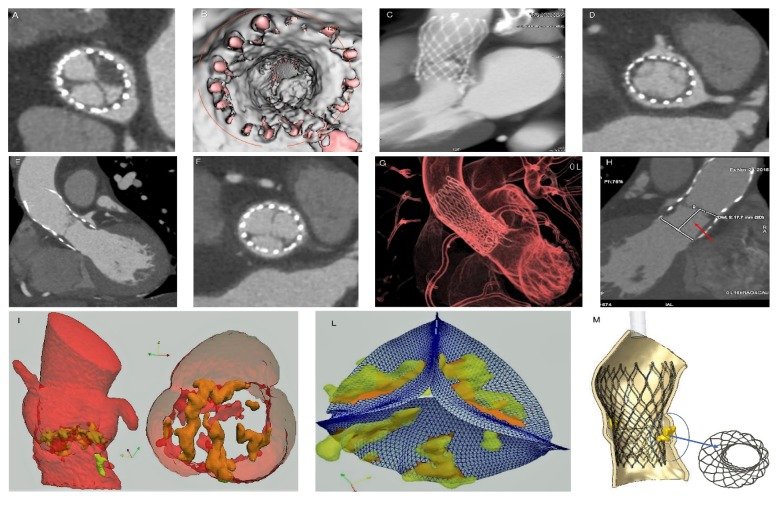
Biomodelling of the TAVR with T HVT and dislodgment. Panels A, B, and C: postoperative CT scan at 1 year shows obstruction of TAVR. The thrombus is located at the level of the posterior and anterior-right leaflet with partial extensions into the supravalvular zone. The posterior leaflet is subtotally fixed in closed position with a planimetry of 1.5 cm^2^. The thrombus is extended to the outer surface of the CoreValve involving the sinus of Valsalva. The coronary ostia were free of lesions and there was no suggestion of embolism. Panel D and E: partial THVT. The thrombus is predominantly organized on the posterior leaflet as a nodular formation (4-4, 5 mm). Panel F and G: CT scan 4 weeks after anticoagulation shows regression of THVT with normal cusps. Panel H: TAVR with THVT and dislodgment. Panel I and L: root and leaflet calcification after extraction through FEA. Panel M: TAVR simulation showing refractory native calcification, stent distortion, and incomplete deployment of core valve 26 mm.

**Figure 5 fig5:**
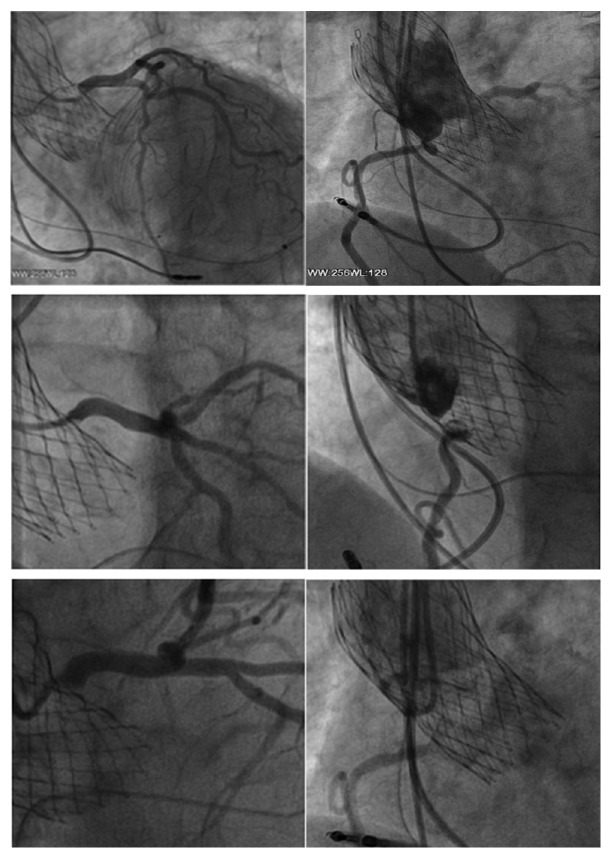
Angiogram revealed ostial stenosis of left and right coronary ostia.

**Figure 6 fig6:**
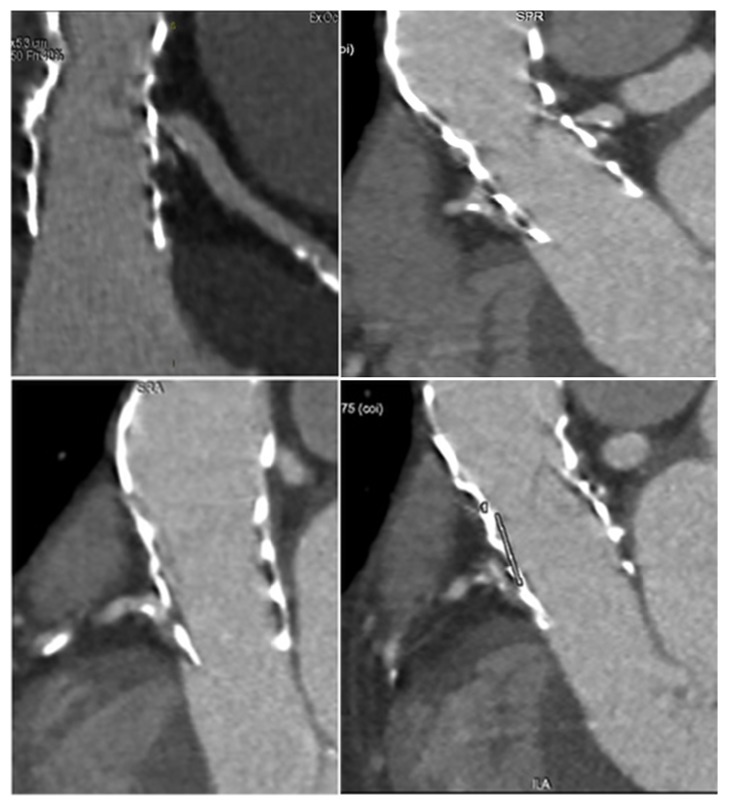
Follow-up gated CT scan demonstrating bioprosthesis malposition with respect to right and left coronary.

**Figure 7 fig7:**
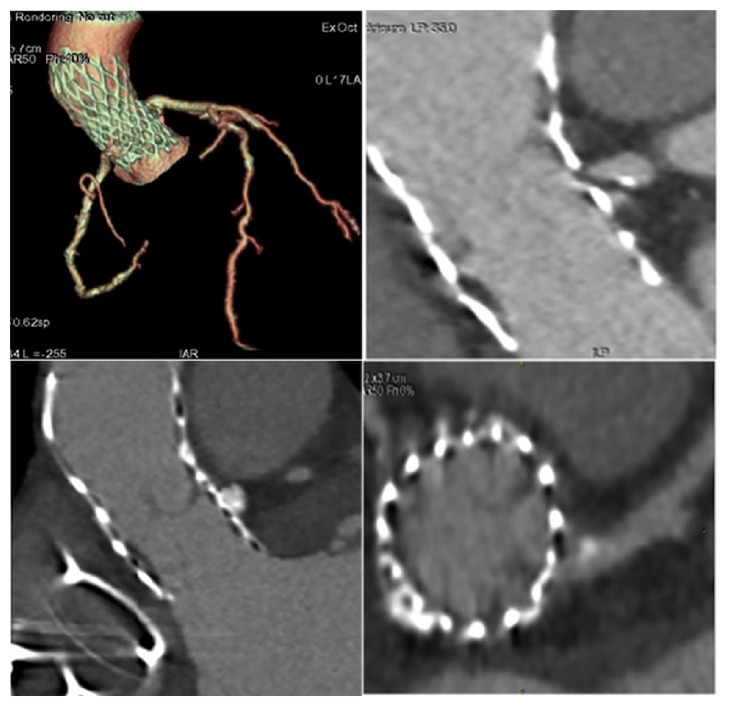
Follow-up gate CT scan demonstrating bioprosthesis migration with respect to left coronary. (S1-S2). Figure 5 S2 upper right: evident presence of the stenosis of the left coronary ostium.

**Figure 8:**
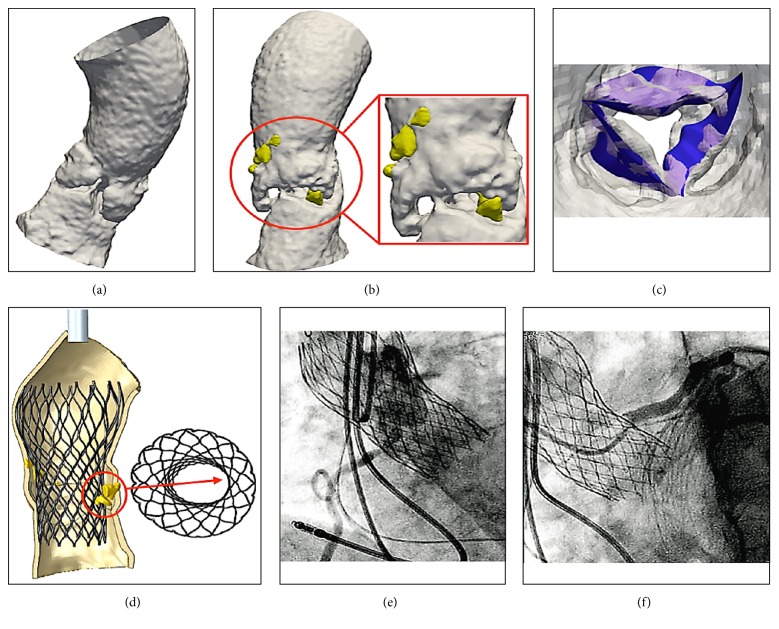
FEA of aortic root and leaflets from CT images after TAVR (a, b, c, d, and e). Calcific blocks and segmentation of blood flow show jagged surface of Valsalva sinus and leaflets (a, b, c). Bulky calcifications determine a noncomplete stent bottom expansion (d). The migration of the device causes the thrombosis of the coronary ostia (e, f).

Three groups of patients who underwent TAVR procedure were evaluated using biomodelling: patients without thrombosis (group 1), patients with THVT (group 2), and patients with THVT and dislodgment (group 3) (Figures 3 [Fig fig4] [Fig fig5] [Fig fig6] [Fig fig7]–8). All recipients were symptomatic for severe aortic stenosis and included in the prohibitive-high-risk group. The baseline characteristics and preoperative data are reported in Table 1. The catheter-based aortic valve implantation was performed using transfemoral access in accordance with established preoperative planning including contrast enhanced CT imaging, which ensures complete information on the size of the ascending aorta, aortic root and aortic annulus for an exact sizing of the prosthesis. Furthermore, CT images were used to assess the suitability of peripheral vessel access to accommodate the relatively large sheaths necessary to introduce the prosthesis. The CT data obtained were compared with preoperative echocardiographic findings. They underwent an uncomplicated transcatheter aortic valve replacement (TAVR) procedure and were implanted with balloon and self-expandable prosthesis. Postprocedural angiography showed satisfactory results with satisfactory positioning and function of the valve (Video 1). Patients were followed up 1-year after procedure. In 546 cases the TAVR showed excellent functioning. 61 patients had thrombus formation of catheter-based aortic valve and 1 patient developed an acute coronary syndrome.

### 2.4. Computed Biomodelling Study

The combined data presented in the case described were used to create an advanced computed biomodelling model. The adopted computational framework to simulate transcatheter aortic valve implantation can be roughly divided into four main phases:

Phase 1: processing of medical images.

Phase 2: establishing suitable models for analysis.

Phase 3: simulation of the entire clinical procedure after analysis of the acquired data.

Phase 4: postprocessing of the simulation results and comparison with follow-up data.

The commercial finite element solver Abaqus 6.14 by Dassault Systèmes (Simulia, Providence, RI, USA) was used to create an aortic valve Finite Element Model (FEM) and perform all simulations (stent crimping and prosthesis implantation in the native root).

Preoperative CT images were used as a starting point to create a patient-specific geometrical model of the aortic valve complex, consisting of aortic root wall, native leaflets, and calcific plaques.

### 2.5. Native Aortic Root Model

ITK-Snap 3.6 software (www.itksnap.org) was used to capture the main anatomical features of interest and generate the three-dimensional reconstructions: the aortic wall surface was extracted from DICOM images by semiautomatic segmentation. The STL representation of the aortic root, outcome of the segmentation procedure, was used as input for an in-house developed Matlab code (v.R2017a, Mathworks Inc, Natick, MA, USA) that allowed the generation of the outer profile of the wall with a constant thickness of 2.5mm, for simplicity. The output file contained Rhinoceros 5.0 commands (McNeel & associates, Seattle, WA, USA) and used to get an IGES file representing the final 3D CAD geometry of the aortic root. The obtained volume model of the aortic root was then imported to Abaqus for the discretization using C3D4 tetrahedral elements.

Native leaflets were geometrically reconstructed and modelled using 4-node shell elements with reduced integration (approximately 7.000 S4R elements) and under the assumption of a uniform thickness measuring 0.5mm [43]. Abaqus was used for the definition of the attachment lines (between the leaflets and the root) and Rhinoceros software was then employed to define the free margin of the leaflets to reconstruct the surface in the open configuration.

### 2.6. Calcifications

In order to accurately reenact the TAVR procedure, calcifications were included. Although attributing specific material properties to a patient's calcifications from CT data was difficult,** calcifications may** drastically affect the efficacy of TAVI procedure [43]. In this case, the software ITK-Snap was used to extract and segment relevant calcium deposits from preoperative CT images, using a threshold of 800 Hounsfield units. The STL file of calcifications was then imported to a software called ParaView v.5.4.1 (open source multiple-platform application for scientific visualization, geometries modification and data analysis) and processed using VMTK (Vascular Modelling ToolKit, www.vmtk.org) to obtain a regular tetrahedral volume mesh. Each calcific block is treated as a single entity and imported as a new part in the Abaqus model. A kinematic coupling constraint technique is used to rigidly connect the surface nodes of the calcium deposits to specific reference nodes of the leaflets. In this way, translational and rotational degrees of freedom of calcifications are suppressed and replaced by those of the leaflets reference nodes.

### 2.7. Prosthetic Model

An accurate geometrical model of the self-expandable Medtronic CoreValve size 26 (Medtronic Inc., Minneapolis, MN, USA) is obtained initially from high-resolution micro-CT images of the actual device** after its expansion**, modelled using Nitinol constitutive laws [44]. The CAD model of stent frame was built using Rhinoceros 5.0 and Matlab to obtain the entire description of the device. Abaqus Explicit was used to compute a finite element simulation of the crimping step. The whole model consisted of two parts: the CoreValve stent and the catheter. The stent frame model was meshed using approximately 70.000 solid brick elements (“C3D8R”) with reduced integration. Leaflets made of pericardial tissue were not included because they did not affect the mechanical stent performance and its interactions with the aortic root wall. The simplified catheter was defined using a cylindrical rigid surface, meshed using quadrilateral surface elements (approximately 10.000 “SFM3D4R” elements) with reduced integration. After crimping the device within its delivery system, the rigid catheter was gradually removed with an upwards sliding movement to allow stent opening, thereby exploiting its super-elastic behaviour, accurately described by the Nitinol constitutive laws proposed by Auricchio et al. [41], both for crimping and expansion simulations.

### 2.8. Material Models

To characterize leaflet tissues, simplified isotropic St.Venant-Kirchhoff material properties were considered, with Young's modulus E of 2 MPa and a Poisson's ratio *ʋ* of 0.45. In order to represent the nearly incompressible nature of the cardiac root tissue, the hyperelastic material model was described by a six-order reduced polynomial constitutive model (material parameters proposed by Martin et al.) [45]. Density *ϱ* was assumed equal to 1.1e^−09^ Tmm^−3^ for both aortic wall and valvular leaflets [37], while the calcified tissues adopted the following parameters: E=10MPa, *ʋ*=0.35, and *ϱ*=2**∗**10^−09^ tonn/mm^3^ [46].

### 2.9. Simulation Details

During the entire simulation, kinetic energy was monitored to ensure the ratio of kinetic energy to internal energy remained less than 10%, so the stent deployment could be considered as a quasi-static phenomenon. To effectively capture the in vivo conditions of the aortic root, preliminary boundary conditions were applied to both its extremities (constrained to a plan normal to the axis of the stent), in order to prevent excessive movements, while nodes at the bottom of the stent were blocked to prevent longitudinal translation of the prosthetic device during the release phase. Self-contact was defined for the stent elements and friction coefficient was set to 0 to model the interaction between the inner surface of the sliding catheter and the stent along with its outer surface and valvular structures. The time period defined to simulate the stent delivering phase from the catheter inside the aortic root was set to 0.4 seconds and a semiautomatic mass-scaling strategy on the CoreValve elements set was used to speed up the analysis and reduce its computational cost. This artificial increase of the mass improves the computational efficiency of the analysis while retaining the necessary degree of accuracy required for this particular problem by forcing the “Time Step” parameter to be 10^−07^ seconds.

## 3. Results

### 3.1. Patients without THVT

At 12 months after TAVR procedure, 546 (90%) were NYHA class 1-2. No thrombotic formation was noted at their CT imaging. Biomodelling showed correct positioning and deployment of the catheter-based aortic valve. There were no stent deformations (Figure 3, Panel A and B).

### 3.2. Patients with THVT

61 patients (10%) had THVT at 1-year follow-up. Preprocedural Gated Computed Tomography (CT) scan showed an Agatston Score Median of 3147 AU (IQR 1997-4352). Pre- and postprocedural features of the aortic root are described in Tables 1 and 2 and Figure 4. Postprocedural CT scans showed thrombosis of the inner surface of devices with thrombotic formation to different extensions located in subvalvular zone (Figure 4, Panel A-F). The largest extension was measured at 24 mm × 18 mm (Figure 4, Panel A-C) while a small thrombus of 4-4, 5 mm and was predominantly organized on one leaflet as a nodular formation (Figure 4, Panel D-E). The thrombus was extended to the level of the two leaflets. The supravalvular zone was involved with one leaflet subtotally fixed in a closed position with variable planimetry. In some cases, the thrombus extended to the outer surface of the device involving the sinus of Valsalva (Figure 4, A-C). In 60 cases (98,36%) the coronary ostia were free of lesions and there was no suggestion of embolization (Figure 4, Panel A-E). A month after initiating treatment, CT scanning revealed a partial extension of subvalvular thrombus with a marked regression of the circumferential supravalvular mass partially confined to one of sinus of Valsalva (Figure 4, Panel F and G) in some patients.

Preprocessing of preoperative aortic root and leaflet model through the finite element analysis was used to establish the geometry of the leaflet and the root. Calcific blocks extracted from CT imaging were also included in the biomechanics model preprocessing for completeness. The stent shape was evaluated for the presence of refractory bulky calcification. We simulated device apposition and anchoring that was compared to postoperative CT scan. FEA simulation of TAVR procedure was performed revealing refractory bulky calcifications after deployment of the self-expanded valve which did not cover the entire circumference of the annulus, resulting in a large paravalvular orifice. The device was not aligned with the aortic root, thus, lacking complete basal attachment and showed stent deformation (Figure 4, Panel I-G).

### 3.3. Patients with THVT and Dislodgment

Delayed prosthesis malposition and stent distortion was noted in 6 TAVR recipients (9,83%) with thrombotic formations. Calcium score index by Agatston score had a Median of 1965 AU (IQR 820-3110). One patient had migration of the device and obstruction of the coronary arteries with a lower calcium score index (820 AU) as well as marked changes in morphology and aortic root dynamics compared to patients without thrombus formation (STJ 28 mm × 22 mm versus 38,5 mm × 36,3 mm; LVTO 26,9 mm HVT) and delayed prosthesis malposition had significantly less aortic root calcification compared to patients with thrombotic formations alone (median Agatston score 1965 AU (IQR, 820-3110) versus 3147 AU (IQR 1997-4351), P = 0.017). Fibrosis and thrombosis of coronary ostia after TAVR were strongly associated with the asymmetric LVOT measurement and STJ with a potential “Bernoulli effect” boosted by implantation and consequential disturbance of kinematic viscosity characteristics (fluid-dynamic component).

Angiography demonstrated ostial stenosis of both the left main stem and the right coronary ostia (Figure 5) which were obstructed by a mild paravalvular leak of the bioprosthesis at TTE and TEE echocardiography. A gated CT scan with 3D reconstruction revealed stenosis of the right and left coronary ostia (Figure 6) and valve malposition with cusps situated 14 mm above the ostium of the right coronary artery (Figure 7). Fibrous and calcific agglomerations were adherent to one of the cusps causing a tight stenosis of the left ostium (Figure 7). The valve was otherwise well deployed and functioned satisfactorily. The patient underwent emergency coronary artery bypass grafting using left internal mammary artery to bypass the left anterior descending artery and right internal mammary for the right coronary artery. Her recovery was uneventful and she was discharged home on the 6th postoperative day. The results of computed biomodelling revealed an Agatston Score Median of 1965 AU (IQR 820-3110) was a predictive factor for valve dislodgement (Agatston score median 1965 AU (IQR, 820-3110) versus 3147 AU (IQR 1997-4351), P = 0.017) after evaluation of patient's aortic root and leaflets through finite element analysis (FEA) simulation of catheter-based aortic valve implantation (Figures 4, 7, 8(a), 8(b), 8(c), and 8(d)).

Firstly, the calcific blocks and segmentation of blood flow, directly extracted from CT imaging, showed a more jagged surface of aortic sinus and leaflets without accentuating calcifications (Figures 7, 8(a), 8(b), and 8(c)) confirming the paucity of calcareous deposits. Second, the persistent refractory bulky calcifications could result an incomplete stent expansion caudally (Figures 7 and 8(d)) highlighting weak anchoring of the device. Both these conditions may cause delayed prostheses malposition.

## 4. Discussion

Knowing the cause of TAVR thrombosis has important implications for treatment and management of severe aortic valve stenosis with balloon-expandable transcatheter aortic valves. Patients with conventional stented xenografts are not routinely treated with dual antiplatelet and anticoagulants unless other attributing factors are present (i.e., previous PCI, atrial fibrillation, etc.). A previous study demonstrated a 7% rate of transcatheter heart valve (THV) thrombosis in a large series of patients undergoing the CT controlled procedure, while 18% of these patients experienced clinically obstructive thrombosis of TAVR [41]. The authors noted that the risk of THV thrombosis in patients who did not receive anticoagulant treatment was higher compared to patients who received warfarin (10.7% versus 1.8%; risk ratio [RR]: 6.09; 95% confidence interval [CI]: 1.86 to 19.84) and larger sized self-expandable transcatheter aortic valves (≥29mm) had an increased incidence of THV thrombosis (p = 0.03).

Valve malpositioning was recognised as another important precursor of complications for TAVR procedures. This is demonstrated by the current efforts in assessing the efficacy of novel devices which are repositionable at the moment of the procedure [46] or designed to prevent coronary obstruction [47]. However, we noted that delayed malposition is often accompanied by a reactive inflammatory/fibrotic process at the level of the migrated cusps leading to calcification and stenosis of the left coronary ostium. To our knowledge this delayed migration and clinical presentation has rarely been described in the literature and is usually limited to the immediate postprocedural in-hospital stay [47–49]. This was also not described in recent reviews [50, 51]. In our case, migration and coronary obstruction presented long after the index procedure. A combination of factors such as the dimensions and anatomy of the aortic root, coronary ostia height, valve dimension and low calcium score may have played a role in this event. A calcium score <2359 AU has been identified as the single predictive factor of valve dislodgement in a recent study [52] which was a similar finding in our study. Although CT scanning allowed identification of the fibrosis / calcification originating at the level of the misplaced valve thereby causing coronary obstruction, computerized tomographic images were unable to predict dislodgment of the device based on calcium index score. In this case, predictive biomodelling in addition to CT scanning was an immensely valuable aid for the diagnosis, clinical decision-making, and management of this patient, which would have otherwise been solely reliant on angiography which would have been challenging. Coronary obstruction related to the insertion in the aortic root of complex expandable devices is one of the most daunting and lethal complications in this context, as demonstrated by the recent development of a device, the ACURATE TA, aimed at mitigating this risk [53–55].

In a recent analysis of the real-life scenario of TAVR in the 2016 Annual Report of the Society of Thoracic Surgeons/American College of Cardiology Transcatheter Valve Therapy Registry, one-year mortality and morbidity continued to be elevated with no consistent advantage over SAVR at 1-year follow-up [56]. More accurate investigations have been advocated [42] for unveiling predictors of negative outcomes and aiding patient selection especially for those who are unlikely to benefit from the procedure due to the risk of complications, survival and quality of life [50]. The use of preoperatively biomodelling integrated with advanced CT imaging in this context should be promoted and supported. Biomodelling through FEA and 3D CT scan imaging might also provide important information for the development of geometrical solutions to predict complications and plan optimal procedural strategy preoperatively [54].

We note that data reported in the literature [56] have rarely focused on specific preoperative measure of LVOT, annulus, aortic sinus and STJ in relation to potential for valve thrombosis. Moreover, no significant data was available concerning the direction of flow and the geometric axis of the expanded valve in relation to the axis of the LVOT, annulus and aortic sinus. Ribeiro et al. warned that a consistent mismatch with dimension of the aortic sinus should raise suspicion for malposition and migration of TAVR that may cause coronary ostial obstruction [53, 57]. However, the relationship between different anatomical components where the valve is placed (LVOT, basal attachments of aortic valvar leaflets, anatomic ventricular arterial junction, crown like ring, sinotubular junction), as well as the mechanical stresses on leaflets and stent with fluid-dynamic characteristics deserve further investigating. Why does a large valve size, especially when used in combination with dual antiplatelet therapy undergo extensive thrombosis causing obstruction? The presence of a strong dynamic fluid disorder associated with an inadequate geometric orientation of the valve (partially distorted) may be the sole explanation. In the case described, there was a double funnel with an important mismatch between LVOT and valve size. The size discrepancy and evidence of a narrow ST junction could have determined the “double hinge effect” in where the CoreValve remained compressed.

The convergence of the data provided by CT scan and biomodelling should be a determining factor for predicting complications after TAVR implantation. This may safely permit its use in the intermediate/low-risk patient.

## Figures and Tables

**Table 1:** Preoperative CT scan methods of severe aortic valve stenosis acquisition data before TAVR.

**Procedure**	**Acquisition**		**Stratification data**	
**CT scan 250 Multislice**	**DPL**		**AORTIC VALVE ANALYSIS**	
Leaflet and aortic root features and behavior	459 mGy/cm	Functional aortic structure assessment	Morphological aortic valve study	Anatomical AVA determination
Aorta CTA and TAVI planning	(i) Fast acquisition (<7 sec) with uniform contrast. (ii) Ensure excellent IQ on coronaries, aortic valves & ascending aorta even without Betablocker	Mixed axial gated & helical ungated modes	Lower dose: up to 70% dose reduction	MMAR to reduce metal artifact when hip prosthesis

**2D Echocardiography**				
Aortic valve leaflet	Parasternal short-axis	>20 mm	96%	97%
Aortic valve area	Parasternal long-axis	≥2.5 cm^2^	64%	95%
Aortic Gradient	Parasternal long-axis	≥39.5°	98%	97%
ITVI	Parasternal long-axis	≥45°	100%	95%
Left ventricular end-diastolic diameter	Parasternal long-axis	>65 mm	Not available	Not available
Left ventricular end-systolic volume	Apical 4-chamber	≥145 mL	90%	90%
FE Teichholz	Parasternal long-axis	≥2.5 cm^2^	64%	95%
Pisa radius	Apical 4-chamber	≥10 mm	64%	90%
Mitral Gradient	Parasternal long-axis	≥11 mm	81%	84%
Basal aneurysm/dyskinesis	Apical, parasternal, or short-axis	Present	Not available	Not available

**3D Echocardiography**				
Aortic leaflet	Full volume modeling	≥29.9°	85%	89%
Aortic area	Full volume modeling	≥29.9° + yes	85%	92%

## Data Availability

The data used to support the findings of this study are available from the corresponding author upon request.

## Supplementary Materials

Supplementary Materials
**Video 1: **TAVR of prohibitive surgical risk. The video shows the success of the implanted CoreValve 26 mm with restoration of adequate surface of aortic area.** Video 2: **1 year later cineangiography shows thrombus formation of right and left coronary ostia.
